# Should more attention be paid to polio sequela cases in China?

**DOI:** 10.3389/fpubh.2022.1076970

**Published:** 2023-01-20

**Authors:** Jiancheng Zang, Longfei Feng, Jichao Wang, Xiaonan Wang, Kun Li, Xiaomei Zhai

**Affiliations:** ^1^Center for Bioethics, Chinese Academy of Medical Sciences and Peking Union Medical College, Beijing, China; ^2^Department of Ethics and Health Policy, School of Population Medicine and Public Health, Peking Union Medical College, Beijing, China; ^3^Aesthetic Medical School, Yichun University, Yichun, China

**Keywords:** polio, disability, rehabilitation, public health, ethics, population medicine

## Abstract

Since “Global Polio Eradication Initiative” was launched by World Health Assembly in 1988, the incidence rate of polio has been reduced by more than 99%, and the whole world has entered a post polio era nowadays. China has been a polio free status recognized by World Health Organization for 22 years and most people believe that no more public health concerns need to be given. How is the population of polio survivors in China? What strategies of health economics and actions of public health for those with polio are ethically appropriate? This article, first of all, deeply summarizes and analyzes the history, current situation and unmet needs of population with polio sequelae and post-polio syndrome in China, and then, puts forward important issues faced by polio survivors who natural infected and who due to vaccine associated paralytic polio and vaccine derived poliovirus. The management of polio survivor is not only a medical and rehabilitation problem involving accessibility, accommodations, but also a public health issue, and most importantly, an ethical concern. Furthermore, from the perspective of ethics such as Justice and Cooperation, the author demonstrates the rationality and necessity of continuing to pay more attention to polio sequela cases at this stage in China. Finally, many valuable suggestions and practical recommendations are given.

## Introduction

Polio is an acute infectious disease caused by the poliovirus (1). Many people infected with polio have flaccid paralysis of their limbs resulting in lifelong disability. This resulted in a lot of affected sequela cases in the last century particularly in the 1950–1970's. Currently, polio has been nearly eradicated globally with extensive vaccination efforts utilizing the Salk and Sabin vaccines and the joint efforts of people all over the world. At present time, most polio sequela cases are 60–70 years old, and those in China are much younger. Although survivors may have disabilities, it is no longer a significant issue in some countries because they are able to function well with good accessibility to treatment over the years. The status of polio sequela cases in China may be some different.

In China, polio has been reported since the 1950's. The number of polio cases was as high as 10,000–30,000 every year during the 1960's according to an incomplete statistical account (2). In view of this severe situation, the Ministry of Health had Gu, who worked in the Chinese Academy of Medical Sciences, lead a team to the Soviet Union to learn about preparation of polio vaccine in 1959. Professor Gu compared the mechanisms of preparing inactivated and live attenuated vaccines. Given the large population in China at that time, it was imperative to reduce the incidence of polio quickly, and the country's economy could not afford the production costs of an inactivated vaccine. Therefore, the relevant departments approved an immunization strategy using a live attenuated vaccine (2). In March 1960, a live attenuated vaccine was successfully developed, and it was officially put into production in 1961. Unfortunately, several small-scale outbreaks occurred in the late 1970's and 1980's, because the preservation, transportation, and distribution of vaccines was a complex social procedure, especially in rural areas. A trivalent vaccine was successfully developed in 1985 and popularized nationwide in 1986, which provided a powerful weapon for the complete eradication of polio in China. With this vaccine-preventable disease controlled (3), and through the efforts of the Chinese government and institutions at all levels, there have been no new polio cases caused by indigenous wild poliovirus (WPV) since October 1994. In October 2000, China was declared polio-free of the WPV by the Regional Commission for the Certification of polio Eradication in the World Health Organization (WHO) Western Pacific Region^1^. With the launch of a domestic inactivated polio vaccine, China have began to gradually promote a sequential vaccination program to prevent polio. On May 1, 2016, a 1+3 program was implemented [one dose of inactivated polio vaccine (IPV) and three doses of oral polio vaccine (OPV)], and on January 1, 2020, a 2+2 program was implemented (two doses of IPV and two doses of OPV) (2). Ultimately, IPV will be used for all four doses.

In the current post-polio era, most people believe that there will no longer be polio in China in another 20 years, and polio sequela cases will no longer be a problem as they will need no further care. Should more attention be paid to polio sequela cases in China? Have medical rehabilitation resources met their healthcare needs? How is the care, health, and wellbeing of individuals with polio provided? What strategies of health economics and actions of public health for those with polio are ethically appropriate?

## The population of polio sequela cases in China

Polio sequela case, sometimes called “polio survivor” because they survived from the epidemic fortunately. According to the official data of the China Disabled Persons' Federation in 1994 (4), Xinming Song's literature published in 2013 (5), and polio sequala chapter of “Lower Limb Deformities” published in 2020 (6), the size of population of polio survivors in China is about 2.0 million from the last century, accounting for about 1/10 of the total number of the people with limb disabled. Most of these people have varying degrees of lower limb disability, joint dysfunction, and loss of muscle strength and mobility of multiple muscle groups (6). Some have implemented rehabilitation regimes and undergone surgeries (7), some have given up treatment, but most have been searching for rehabilitation and other solutions. They are gradually entering middle-age or have become elderly (8). They often manifest complex symptoms as limb weakness, fatigue, cold intolerance, joint and muscle pain, aggravation of original deformities as well as some new deformities, such as dysphagia, oral motor weakness, and paralysis. These are referred to as post-polio syndrome (PPS). It is estimated that 50% −80% of polio sequela cases suffer from PPS which generally occurs 30 years after the acute onset of polio (9). The demand for surgery and rehabilitation in this population is not disappearing but increasing with disability prevention and social progress, because these syndromes will aggravate their existing movement disorders, reduce their mobility, and affect their life, marriage, family, and work. Polio sequela cases has the distinctive characteristics, which is constantly changing, as well as the demand for medical treatment and rehabilitation during the whole life span.

## Unmet needs of polio sequela cases in China

### Most healthcare institutions oppose paying attention to this shrinking population

Since the 1980's, the polio epidemic has gradually been effectively controlled globally. The last case of polio in the United States was reported in 1982. The last case of indigenous WPV in China was in 1994, and just 4 cases of WPV importation into the entire country have been reported since 1995 with the last case in 2011. Considering the large number of polio survivors, 10 authorities of the Chinese government, including the Ministry of Civil Affairs, the Ministry of Health, the State Planning Commission, the Ministry of Finance, and the China Disabled Persons' Federation jointly deployed and implemented the national program named “Three Rehabilitation,” one of which is polio deformity correction, in September 1988. By the end of 1993, 327,688 patients with polio had received treatment, with an effective rate of 98.7% and a remarkable rate of 81.1% (4). Due to the success of the national immunization program, the number of new polio cases is getting smaller and smaller, Medical treatment and rehabilitation of polio has never been listed in special regulations and national programs again. In order to adapt to changes of the disease spectrum, the original orthopedists, who primarily focused on correcting deformities, were laid off or transferred to other positions when China entered its polio-free status. Hospitals no longer establish polio specialist clinics, and medical colleges or universities have no institutions addressing polio. Concurrently, medical conferences rarely address this issue, and related scientific research is seldom to be found.

### Insufficient medical community awareness of polio sequelae and PPS

Once the end of the acute phase of the polio epidemic occurred, people believed that the patient's story was over, and they received their immunizations against it. However, the issue of polio sequela and PPS is occurring right now (10). The clinical manifestations of polio sequela are complex and diverse. It is difficult for inexperienced doctors to distinguish PPS from common diseases of the elderly, and its treatment does not follow a common routine. There is a lack of research on PPS, and patients with PPS are often accused of hypochondriasis. Doctors who are rich knowledge of polio sequela have retired, and there are few institutions engaged in limb disability correction. Most medical staffs and rehabilitation practitioners have little awareness of PPS, and they have a poor understanding of the process for polio care. Furthermore, relatively few doctors and institutions can provide appropriate diagnosis, surgical care, and rehabilitation. As a matter of fact, the existing medical rehabilitation and policy system do not match with the specific needs of polio population, because the development of PPS raises questions of polio as a static disease and it poses a challenge not only to health professionals, but also to policy makers who are responsible for providing the necessary health care measures and corresponding resources (11).

### Medical science popularization and disability education for PPS lags behind

Although previous rehabilitation interventions focused on overcoming disability at all costs, much evidence show that the original deformities from polio are aggravated, or new deformities appear with aging. Good results have been achieved in treating these complications through rehabilitation intervention and orthopedic surgery (12). Even though most people with disability still have difficulty in adapting to the environment and realizing “normalization,” the rapid development of new medical technology [exoskeleton, artificial intelligence, and advanced limb reconstruction technology (13)] can greatly improve previously untreatable physical disabilities. However, polio survivors, especially those in rural and remote areas, have a limited awareness of polio itself and PPS due to the relatively low level of medical services and their different culture. There is little space in medical textbooks currently that discuses polio and its sequela. The dearth of education for medical practitioners caring for those with physical disabilities restricts the possibility of quality-of-life improvement for polio survivors among the middle-aged and elderly in China. The lack of access to diagnostic and treatment information for these patients leads to worsening of their own complications, and there is no access to rehabilitation, surgery, and other improvement measures.

### Management of cases with vaccine related paralytic polio and vaccine derived poliovirus need more attention

The WHO emphasizes that vaccine related paralytic polio (VAPP) and vaccine derived poliovirus (VDPV) are unavoidable adverse events of vaccination. This is a significant issue with the live attenuated vaccine, and occurs with an incidence of 2–4/1 million newborns (14). They cannot be ignored. In 2019, the Chinese government promulgated *the Vaccine Administration Law of the People's Republic of China*^2^. It formulated relevant policies to help those who experienced these unfortunate complications and established corresponding compensation for those identified with VAPP. However, whether it can meet the requirements of health economics and meet the health and wellbeing of these individuals' entire lifespan, and how to evaluate the relevant departments have effectively investigated and followed-up patients with VAPP and fully implemented the corresponding policies requires further research (15).

### Common issues of people disabled faced by polio sequela cases

Polio sequela cases with disabilities in China still face issues including, but not limited to, prejudice, stigmatization, personal dignity, autonomy, privacy, fairness, and active participation in society. In fact, disability of polio survivor and others, such as person with cerebral palsy and motor accident, are similar, but the main difference is that there are various types of physical disabilities, full of complicated changes in different life periods, and lack of corresponding medical treatment and rehabilitation. How to protect equal health rights and promote medical research, how to overcome issues in the provision of public health, and how to ensure the accessibility and affordability of medical treatment, rehabilitation, and assistive devices are still questions. These interests must be balanced against with the need for increased public knowledge and debate concerning disability. It is not just a task for healthcare practitioners and providers, also an important challenge faced by all stakeholders.

## Analysis of the reasons

### Systematic barriers may have formed

The government of China has promulgated a number of laws and regulations related to people with disabilities such as *the Law of the People's Republic of China on the Protection of the Disabled, Regulations on the Prevention and Rehabilitation of the Disabled, Regulations on the Employment of the Disabled, Regulations on the Construction of an Accessible Environment, Implementation Opinions on Further Promoting the Civilized Practice of Helping the Disabled, and National Action Plan for Disability Prevention (2021–2025)*. The Disabled Persons' Federation and the Ministry of Civil Affairs have set up a special rehabilitation hospital for the disabled. However, there is no specific description of the special needs of the disabled with polio, and they are not accurately identified, perhaps for the reasons of avoiding discrimination.

Thirty years ago, 10 national authorities jointly implemented the program “Three Rehabilitation” one of which has been dedicated to the treatment and rehabilitation of polio cases for five consecutive years and has achieved great success. After the completion, the “Three Rehabilitation Office” was renamed the “National Rehabilitation Office for the Disabled” (4), because the goal is to take care of all the disabled, not only for polio. Unfortunately, the attention paid to the polio population has gradually decreased with the changes. The disadvantages brought about by the fragmentation of information among various authorities have emerged. It's a pity that no national action on polio has been held thereafter. As the time goes on, there are less and less hospitals or departments that can provide treatment and rehabilitation for polio survivors, just only one hospital of the Ministry of Civil Affairs right now. Even for that other disabilities, such as person with sequela of cerebral palsy and trauma event can be treated and recovered in many hospitals.

The healthcare institutions affiliated to the Ministry of Health do not set up a department for polio deformity correction and rehabilitation. The number of doctors familiar with the polio sequela and PPS is limited. The disabled usually go to hospitals first when they need help, while they does not know about disability so much owing to just little space in textbooks. Therefore, polio sequela patients usually cannot receive adequate medical rehabilitation and treatment. For polio cases in rural and remote areas, it is more difficult to meet their medical needs. System barrier directly lead to the rehabilitation of the disabled is separated from Health and Education.

### Polio survivors in China need additional helpful experience

The formulation and application of many intervention strategies have played an important role in polio control and prevention worldwide. Among these are the Strategic Plan for the Final Phase of Polio Eradication (2013–2018) (16) the Strategic Plan (2019–2023)^3^ and the Polio Eradication Strategy (2022–2026) (17). All of these plans note that polio eradication requires the eradication of cases caused by WPV as well as VAPP and VDPV. As new cases occur in Pakistan (18) Afghanistan (19) and New York (20) there are many reports issued by those who pay attention to and promote the Global Polio Eradication Initiative (21). These reports focus more on the promotion and use of vaccines and virus surveillance (22). Only rarely are policy recommendations for the management of those who have survived polio espoused (23). The elimination of poliovirus is important and well-known, but it does not mean eliminating patients surviving polio. According to the International Classification of Functioning, Disability and Health (ICF), appropriate strategies for the treatment and rehabilitation of those surviving polio are necessary.

Due to the time of prevalence and control of polio epidemic, their population in China is relatively young. Those surviving polio with disabilities in different regions of China have varying needs for deformity correction, education, medical treatment, social environment, and economy. They should still be paid more attention to although the number of polio cases is decreasing. In particular, many countries have polio organization, such as *March of Dimes*^4^*, Polio Canada*, and so on, which are accurately guided and organized, making rehabilitation education more accessible. Furthermore, polio clinic, a specialized setting, can clearly organize professionals to take charge of prevention, diagnosis, control, treatment, rehabilitation and health promotion for polio cases. This is what the Chinese group of polio should learn from and vigorously promote and expand. Importantly, Chinese Disabled Persons' Federation should also pay more attention to this relative mature experiences of rehabilitation education, which were the weakness of Chinese polio, and strengthen its guidance and promotion.

The reduction of new polio cases, the formation of barriers between authorities, and the lack of communication between different regions and countries may be the main reasons for the current situation of polio sequelae and their unmet needs. In determining a framework to provide care for polio sequelae cases in China, polio eradication should not be the only goal (24). Early and effective intervention are of great significance for improving their quality of life and social integration. Learning more from international experiences on comprehensive and system governance and considering how to better implement a strategy for the specific populations in China is imperative. It is essential to build an appropriate framework for the entire lifespan of individuals with polio and provide multidimensional care for this whole population.

## Ethical consideration

How is it possible to identify the ethical dilemmas faced by polio sequelae cases with physical disabilities? Should we just give up paying more attention to these individuals in China? What strategies of health economics and actions of public health for those with polio are ethically appropriate? It is not only a medical and rehabilitation problem but also a public health concern, and most importantly, it is also an ethical issue.

### Justice

This is a misunderstanding for the discussion of needs of polio sequelae cases based on Bentham's philosophy, which may be another important reason why the polio survivors were not given enough attention in reality. It is generally believed that giving priority to the cost-effectiveness of health care resources may lead to the lower priority of treatment for the disabled than other similar non-disabled people. The defenders of cost-effectiveness insist that the cost-effectiveness of treatment for the disabled is low. If we give priority to non-disabled people, we will get greater health benefits. This kind of Utilitarianism is generally held to argue against special attention for the disabled as Dan W and Brock mentioned in their article (25). It implies that disabled persons' lives are of lesser value than those of non-disabled persons. It is disability discrimination. The core value of social public policy should focus on the vulnerable groups firstly. It is injustice if a patient has a lower priority to receive treatment due to his/her illness and disability than others (26). The life value of polio should be respected as well as that of other disabled people. Any disability, no matter how slight or serious, does not mean even worse in political or moral status.

As a vulnerable population, disabled polio survivors need more attention. The option of giving up and waiting for them to naturally disappear cannot be defended ethically. The development and dissemination of helpful medical rehabilitation technologies should be given priority, and more resources and information for polio survivors in different regions should be considered in policy formulation. The relevant diagnosis, treatment, and rehabilitation systems should not be subject to market regulation and excessive influence, otherwise, “the value of medicine” will be devalued, and inequality among people will be aggravated. Nowadays, the government or relevant departments should play a key role in the reasonable allocation of resources, which can be justified by ethics, even if it is different from the calculation results of health economics.

VAPP and VDPV cases are unavoidable adverse events of vaccination. It is not an issue of either getting or not getting polio vaccine, or vaccine effectiveness or ineffectiveness, but an issue of the relationship between individual benefits and public interests. Individuals with VAPP/VDPV face many issues. Their ability to work, provide self-care, mobility, learning ability, and social ability are affected to varying degrees. The progression of their disabilities, in particular with PPS, constitute a heavy social burden. This small group should receive more attention for the implementation of compensation policies by the relevant national authorities following ethical principles. This will ensure the demonstration of national responsibility and further avoid and reduce vaccine hesitation.

### Cooperation

Regarding the consideration for the Barriers, it refers to the information barriers and isolated islands between different authorities, disciplines, groups or countries.

For a long time in China, the diagnosis, treatment and rehabilitation of the disabled are managed by the Ministry of Civil Affairs and the Disabled Persons' Federation, instead of the Ministry of Health. The original intention is to provide better welfare care for the vulnerable population, and a lot of works and achievements have been done. Undoubtedly, such system have achieved better welfare care for disabled persons with disabilities under China's social and economic conditions. With the time changes, such institutional arrangements also exposed some subsequent shortcomings. The prominent problem is that there are “Barriers” and “Splits” between the medical institutions of the Ministry of Civil Affairs and the Ministry of Health, resulting in limitations in medical education, training of medical professional, dissemination of rehabilitation knowledge, etc.

Relevant authorities and disciplines should cooperate and work together to make top-level design, consequently, to break down all the barriers. It is important to learn from helpful experience and not sacrifice or neglect any rights or any opportunities of polio survivors for medical rehabilitation, correction of surgical deformities, and functional reconstruction (6). Looking at international disability prevention action over the past three decades, it is clear that there is increasing awareness that disability is the result of the interaction between people and their environment. Accessibility in every part is an important and universal consideration for society as a whole. Accessibility of polio sequela cases to public facilities and the entire sociocultural environment require the joint participation of everyone, and the formulation of effective intervention plans and methods is necessary.

In addition to the above two important ethical principles, we still need to take into account the basic principles, such as “Beneficence” “Respect”, when discussing ethics and governance of the population of polio sequela cases in China.

## Policy recommendations

As for ethical governance and policy recommendations, the Chinese government and authorities at all levels have done a significant amount of rewarding work. However, the problems and issues have not been completely solved or integrated into the medical rehabilitation system and the three-level prevention domain of the public health system. We should pay more attention to polio sequela cases in China. There are currently efforts to put forward and supplement defensible ethical governance suggestions for the healthcare provider, medical professionals, scientific researchers, and policy makers.

First of all, with a view to promoting Justice through improving the construction of the disabled medical rehabilitation system, we propose to establish polio clinic (27) to focus on solving the health and wellbeing related problems of polio population especially for whom with PPS. Moreover, a special team should be set up within hospitals' existing operating departments to undertake the diagnosis, treatment, rehabilitation, and orthopedic needs, science popularization and scientific research, the provision of professional knowledge, regular evaluation of the people who could benefit. We should not only focus on polio vaccination for disease prevention, but also pay more attention to the quality of life of those polio survivors. The needs of all of those with polio should be taken into account, and customized intervention measures should be actively developed. In addressing greater accessibility, research, and better awareness, the rational allocation of medical resources will be promoted, and a comprehensive national and regional system for polio clinics will be well-designed and established to avoid resource waste.

Secondly, in the light of principle of Justice, the implementation and revision of VAPP and VDPV compensation strategies based on the effective research of Implementation Science and Economics of Health (28) and highlighting the responsibilities of government and society should be strengthened. To maximize the health and wellbeing of individuals with VAPP, and minimize the negative impact on individuals and this entire population, an appropriate effective governance framework is required. A continuous dialogue between patients, public, professionals, ethicists, and policymakers is essential.

Thirdly, Authorities of Ministry of Civil Affairs, the Disabled Persons' Federation, the Health Commission and the Education Commission can work together to break down the barriers, promote rational allocation of resources, and facilitate the medical treatment of the disabled, the training of doctors, and the compilation of teaching materials. With the concept of Population Medicine (29), system barriers and information islands will be broken among the five groups of “patients” “professionals” “disciplines” “authorities” and “ordinary.” All activities should be under girded by the conceptual frame-work of ICF, and must be inclusive of the health and wellbeing of people with disabilities at individual level and population level.

The fourth, with the promotion of the Disabled Persons' Federation and the Ministry of Civil Affairs, we suggest to establish a registration system for polio cases, provide them opportunities for the development of rights such as targeted treatment and rehabilitation interventions as soon as possible, and restart the national polio rehabilitation action when appropriate. Along this way, the network work of polio organizations or alliance in China (24) should be actively promote to improve awareness of polio population, to make health information accessible, like similar group in Canada, United Kingdom, United States, and other countries who always hold regular activities and meetings to popularize relative scientific knowledge.

It is gratifying to note that China has launched a great program “University of Rehabilitation Science” with huge investment. More importantly, it is a joint work organized by the National Disabled Persons' Federation and the National Health Commission. Undoubtedly, it is a good news for Chinese polio sequela cases, and an important measure to promote improvement of their unmet needs. We are full of expectations, especially on the top-level design.

## Conclusions

In the 20 years since China became polio free, the population of polio sequela and those with PPS still face many important issues that have been neglected especially in medical treatment and rehabilitation. It is important to concentrate on the individual and population who with polio at this stage from the ethical perspectives of Justice and Cooperation. Practical actions of public health based on the actual situation in China need to be taken to break down the barriers, to solve these unmet needs, to accumulate valuable experience for polio cases, and to refine Chinese wisdom for polio survivors around the world.

## Data availability statement

The original contributions presented in the study are included in the article/supplementary material, further inquiries can be directed to the corresponding author.

## Author contributions

JZ and XZ conceptualized and supervised the paper. XZ provided project administration and resources. JZ wrote the initial draft. JW, XW, KL, and LF revised this draft. All authors contributed, reviewed, and edited the final manuscript draft.

## Footnotes

^1^ https://www.who.int/china/health-topics/poliomyelitis-(polio)

^2^ http://en.npc.gov.cn.cdurl.cn/2019-06/29/c_674711.htm

^3^ https://polioeradication.org/who-we-are/polio-endgame-strategy-2019-2023/

^4^ https://socialpresskit.com/march-of-dimes
